# COVID-19 and Bangladesh: Challenges and How to Address Them

**DOI:** 10.3389/fpubh.2020.00154

**Published:** 2020-04-30

**Authors:** Saeed Anwar, Mohammad Nasrullah, Mohammad Jakir Hosen

**Affiliations:** ^1^Department of Medical Genetics, Faculty of Medicine & Dentistry, University of Alberta, Edmonton, AB, Canada; ^2^Experimental Oncology, Department of Oncology, University of Alberta, Edmonton, AB, Canada; ^3^Department of Genetic Engineering and Biotechnology, School of Life Sciences, Shahjalal University of Science and Technology, Sylhet, Bangladesh

**Keywords:** COVID-19, novel coronavirus, testing, healthcare, community mitigation, non-therapeutic intervention, infection prevention and control

## Abstract

As the coronavirus outbreak quickly surges worldwide, many countries are adopting non-therapeutic preventive measures, which include travel bans, remote office activities, country lockdown, and most importantly, social distancing. However, these measures face challenges in Bangladesh, a lower-middle-income economy with one of the world's densest populations. Social distancing is difficult in many areas of the country, and with the minimal resources the country has, it would be extremely challenging to implement the mitigation measures. Mobile sanitization facilities and temporary quarantine sites and healthcare facilities could help mitigate the impact of the pandemic at a local level. A prompt, supportive, and empathic collaboration between the Government, citizens, and health experts, along with international assistance, can enable the country to minimize the impact of the pandemic.

## Introduction

With the outbreak of novel coronavirus-2 (nCoV-2) declared a pandemic and an international public health emergency by the World Health Organization (WHO), the entire world is working to address it. It is a rapidly evolving and emerging situation. In <5 months after the first emergence of the virus in December 2019, nearly two million people in 185 countries around the globe have been identified as confirmed cases of coronavirus disease 2019 (COVID-19) (1). Researchers across the world are working hard to understand better the biology of nCoV-2 and the epidemiology of the novel coronavirus disease-19 (COVID-19). The estimated basic reproductive number of the virus is significantly higher than many other infectious diseases, and this can potentially result in the capacity of health facilities becoming overwhelmed, even in the countries that have the most developed healthcare systems (2). An estimated 20% of cases lead to clinically serious and complex conditions. With some sporadic cases of serious illness in younger individuals, adults >60 years of age and with co-morbid conditions make up the most vulnerable group.

There are as yet no vaccines or antiviral drugs approved for the disease, and hence, non-therapeutic interventions to control the spread of the virus are the most effective measures to control the disease (3). Worldwide, billions of people are staying at home to minimize the transmission of the virus. Many countries are adopting preventive measures, e.g., remote office activities, international travel bans, mandatory lockdowns, and social distancing. Bangladesh, a lower-middle-income country and one of the world's most densely populated areas, is struggling to combat the spread of the disease. In this write-up, we briefly articulate the current scenario of COVID-19 in Bangladesh and provide some recommendations on how the country can combat this pandemic.

## Bangladesh's Response to COVID-19

**Figure 1:**
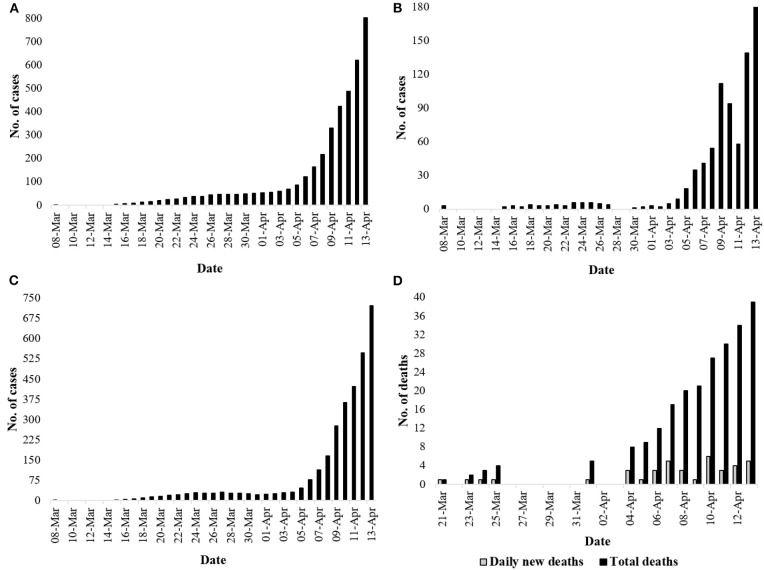
Current situation regarding COVID-19 in Bangladesh (April 13, 2020). **(A)** Total number of cases identified in Bangladesh (5, 6); **(B)** number of cases identified daily (5, 6); **(C)** number of actives cases daily (5, 7); **(D)** number of daily death incidences and total deaths (5).

With almost every country adopting aggressive non-therapeutic measures to control the spread of nCoV-2, Bangladesh in Southeastern Asia has followed the same trend; however, there is an ongoing debate as to whether measures have been adopted adequately and implemented efficiently. The country confirmed the first COVID-19 case in its territory on March 7, though many experts speculated that nCoV-2 may have entered the country earlier than that but had not been detected due to inadequate monitoring (4). As of April 13, the country had reported 803 cases of COVID-19, and the death toll stood at 39 (Figure 1) (5–7). However, concerns have been raised that extreme insufficiency of testing assays may be leaving many cases undetected in the country. In response to the emergence of the virus, Bangladesh admittedly reduced international flights, imposed thermal scanner checking, and shut down schools; however, offices maintained their regular schedules until March 26.

On March 15, the country banned all flights coming from Europe except the United Kingdom; however, the authority still allowed flights from Europe to land in an airport (8). As a result, over 631 thousand people entered the country in just 55 days from January 21 (9). Although the Institute of Epidemiology, Disease Control and Research (IEDCR) claimed that it tested every single person who entered the country, there has been intense criticism of the testing facilities in the ports of entry (10, 11). Beginning on March 16, the country imposed a 14-day obligatory quarantine to all travelers who entered the country (12). It attempted to bring travelers coming from Italy—which was then declared a new epicenter of the pandemic—to a quarantine site. The move was sharply criticized due to a lack of arrangements, and the travelers were allowed to enter the country by themselves on the condition of 14-day-long self-isolation. Since then, hundreds of expatriates who came from COVID-19-affected countries have been seen out in the streets and gatherings—traveling to tourist sites, meeting with friends and families (13). On March 19, the country deployed the army to supervise two quarantine facilities in Dhaka (14).

From the first week of March, Bangladesh started to postpone all mass gatherings, including the 100th-anniversary celebration event of the birth of its founder, Sheik Mujibur Rahman, as a preventive measure against the spread of nCoV-2 (15). Despite these measures, tens of thousands of people gathered in a special prayer session for protection against nCoV-2 in Lakshmipur, despite not having the local Government's permission. Afterward, the Government banned all political, social, cultural, and religious rallies and gatherings in the country (16). Amid this crisis, the country witnessed voting in three constituencies, where people had to go to the voting centers in person to cast their votes. Meanwhile, the health ministry said that nCoV-2 has spread to the community transmission level (17).

Bangladesh admittedly has a severe shortage of testing kits: it does not have more than 100 thousand testing kits in stock, of which only some 20 thousand have been distributed to different testing facilities around the country (9, 18). The country received some testing kits, PPE, masks, and infrared thermometers from China to deal with the crisis in the country; however, this amount only covers a small portion of the country's actual needs (19). In the meantime, utilizing the rapid dot blot technique, Ganashystha Kendra (a local health institution), claimed that it had developed a testing kit that can detect nCoV-2 in several minutes for just BDT 350 (~4 USD) (20). Although many experts questioned the efficiency of the method the kit uses, the institution has reportedly obtained government approval to import raw materials to mass-produce the kits. It is worthy of mention that a very similar rapid testing kit developed and marketed by a Canadian company, which received approval in some Asian and European countries, was refused approval by the health authorities of Canada on the grounds that it may produce a high rate of false-negative results (21).

On March 25, Bangladesh declared the enforcement of lockdown for 10 days effective from March 26. With the enforcement of this lockdown, travel on water, rail, and air routes is banned and road-transportation is suspended. All non-essential organizations, businesses, and educational institutions are closed, except for pharmacies, groceries, and other unavoidable necessities. Following the declaration, many people from the major cities, especially from Dhaka, started to leave the city by various means, including overcrowded public transport services, with a high risk of contracting COVID-19 and in violation of the government instructions. On the same day, Bangladesh issued a temporary release to its ailing former prime minister from prison, and consequentially, thousands of political followers greeted her in Dhaka, defying the lockdown imposed by the Government (22). It was predictable that on the release of a political leader of her fame, a huge gathering might occur; however, she was temporarily released on humanitarian grounds (22, 23).

On March 2 and 3, when the initial 10-day-long lockdown measure was about to be completed, thousands of service and factory workers started heading back to major cities, e.g., Dhaka, Narayanganj, Gajipur, and Chittagong, ignoring the risk of nCoV-2 spread (24). The country's efforts to reduce the spread of the virus in Bangladesh suffered in their implementation due to the lack of coordination between different authorities and groups (24). Later, in two instances, the country declared extensions of the nationwide lockdown, keeping it in place through April 25 (25, 26), and these people coming from different areas of the country had to head back to their home residences (24). On April 5, the country announced a suspension of all international travel except flights to and from China until April 14 (25). It also declared that, as of April 9, some 60 areas of the country, with half of the places in the capital city, would be under a specialized form of localized lockdown to fight the spread of COVID-19. A specialized lockdown was also imposed on Cox's Bazar, a southern district of the country where many Rohingya refugees live (27). These Rohingya refugees, as well as older individuals anywhere in the country, constitute the most potentially vulnerable groups to virus infection.

## Social Distancing Protocol is Tough to Maintain in Many Areas of Bangladesh

As mentioned earlier, Bangladesh did not impose any strict protocol initially, and millions of people were out on the streets, especially in Dhaka, which is a megacity with 46 thousand people living per square kilometer (28). It appears that social distancing is tough while taking public commutes and living in the slums. In the context of massively populated and lower-middle-income countries like Bangladesh, enforcement of social distancing—as recommended by the WHO to stop the nCoV-2 spread—sounds fancy but impractical. Indeed, staying at home is unlikely to be as effective here.

Dhaka, the capital of Bangladesh, is alone home to some 1.1 million slum dwellers (29). These slum dwellers, most of whom have never gone to school and currently live in extremely close quarters, are hardly aware of the threat from nCoV-19. The range of household earnings of slum dwellers in Dhaka is around BDT 8,000/month (<100 USD/month), and they spend >70% of their earnings on food and housing (30). Even a 400-mL bottle of hand soap per slum, which costs around BDT 80 (~1 USD), is hard for them to afford. Besides, every 10–16 families have access to only one bathroom/toilet, where there is no regular supply of water (30, 31). Along with the slum dwellers, Bangladesh also hosts over a million Rohingya refugees, most of whom are living in close quarters in refugee camps where the sanitization facilities are even scarce (32). Fear of COVID-19 is already gearing up among the displaced people in these camps. Immediate enforcement of social distancing is, in every way, practically impossible in a country like Bangladesh.

## Inadequacy of COVID-19 Testing Facilities

**Figure 2:**
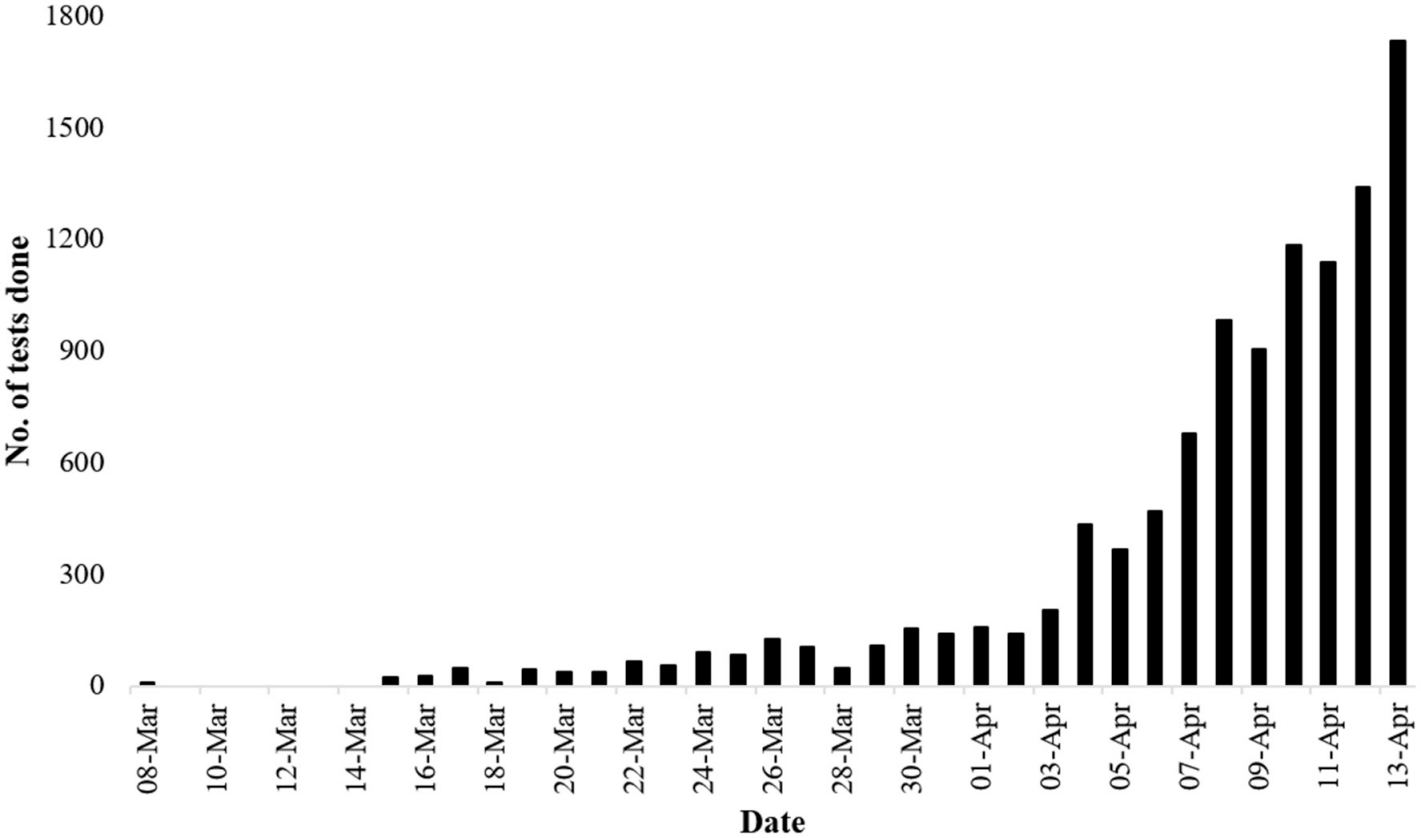
Number of tests done daily. The rate of testing rose significantly 4 weeks after the identification of the first case of COVID-19 in the country (5).

Five weeks after the detection of the first COVID-19 case in Bangladesh, the IEDCR had only tested 11,223 people, constituting approximately 68 tests per million population (5, 7) (Figure 2). It is perhaps among the worst-ranked countries for nCoV-2 testing rate, though the mortality rate is comparatively higher (7). It should be noted that in the first 3 weeks after the detection of the first COVID-19 case in Bangladesh, the IEDCR was the sole diagnostic facility in the country of 180 million people, and the daily testing rate remained below 100 per day (33). The centralization of COVID-19 diagnosis facilities is somewhat plausible, as most hospitals do not have enough personal protective equipment (PPE). However, this left the mass of people and healthcare workers in an awfully susceptible condition. As a result of the combined lack of PPE and diagnostic testing capacity, fear, and anxiety geared up among the mass population, and many healthcare workers refused to provide any service. With much criticism from different sectors, the health authorities of the country ultimately decided to expand its testing numbers from April 3 (33). Currently (April 11, 2020), there are 17 labs across the country working on testing probable/referral cases of COVID-19, and a few more labs are being established in different districts, including one in Sylhet at Shahjalal University of Science and Technology (34, 35).

The situation became even complicated as four doctors at the Dhaka Medical College and Hospital, the largest hospital in Bangladesh, were sent into home quarantine after they handled a person who was later identified as having COVID-19. Later on, many more doctors and health workers were sent into quarantine, and many of them tested positive for COVID-19 (36, 37). The health system of Bangladesh depends on around 100 thousand registered doctors, and if these very few doctors compared to the population size are unable to provide their healthcare service as a result of the unavailability of PPE, this could have potentially catastrophic consequences.

## Mitigation Measures to Fight COVID-19 With Limited Resources

**Figure 3:**
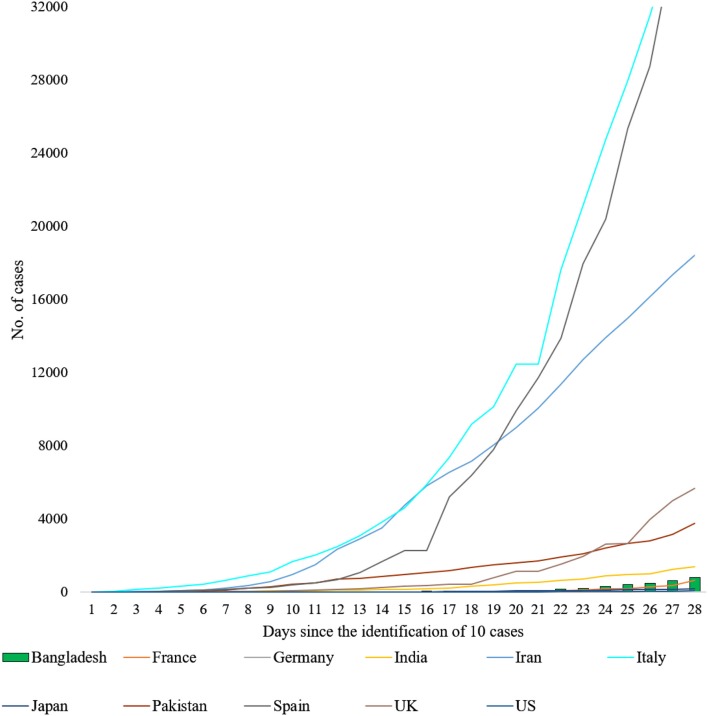
Total number of COVID-19 cases daily after the identification of the 10th case in Bangladesh and in 10 other countries (1, 7). Bangladesh's trendline is comparable with the trendlines of France and Japan. As of now (April 13, 2020), the trendline of the US remains far lower (74 total cases on day 28) than Bangladesh's until the 28th day after the identification of 10 positive cases.

The situation in Bangladesh is rapidly evolving, and it is comparable with many other countries, e.g., France, Japan, which have lately seen a devastating impact from the virus (Figure 3) (1, 7). In this situation, most sensible governments would opt for a total lockdown for an undeclared time at very high financial costs under the precept that lives should be saved first, and counting the loss to businesses may wait. Some countries, e.g., Italy and Spain, have already adopted such measures (38, 39). In fact, with no effective therapeutic strategies available for COVID-19, lockdown is perhaps the best-known measure that could mitigate the situation (40). However, in Bangladesh, where a significant proportion of the total population lives hand to mouth, lockdown is not a feasible idea. With no savings and work, how will poor and marginal people feed themselves if there is a prolonged lockdown? This is an issue that the Government must address when declaring any lockdown or emergency that may stay in place for 2 or more weeks. With help from the armed forces, the Government may think about starting a “hygienic” rationing system in case of locking down for a more extended period.

Among the preventive measures for COVID-19, including aggressive tracing of cases and contacts, strict quarantine, and screening, as well as education to promote good hand hygiene practices, should be put in place (41, 42). Immediate expansion of testing labs to every district and major localities is urgently needed to test every patient with symptoms, and millions of testing kits are necessary for conducting aggressive detection of cases (18). Students at life science departments in universities can be trained to carry out COVID-19 case diagnosis. The molecular genetics, biochemistry, and molecular biology labs in the universities and medical colleges across the country should be quickly transformed into COVID-19 case detection labs. The country can also seek help from China and South Korea on how it can channel extensive detection surveys (43, 44). With help from the armed forces and trained volunteers, the schools could be turned into quarantine centers. The Government will have to come forward to make sure that its marginal population has access to proper hygiene, maybe by supplying free sanitizer and mobile washrooms. All offices and businesses, except medical centers, pharmacies, and groceries, should remain closed until the situation mitigates. Home office laws should be imposed, whenever possible.

Additional measures must be taken promptly, anticipating the potential challenge that would be faced by the hospitals in the case of an upsurge of COVID-19 cases. The Government must source enough protective gear for the healthcare workers who will have to tackle COVID-19 patients in the frontline. With expert help from China and South Korea, Bangladesh should immediately organize specialized training for all physicians, resident doctors, and intern doctors.

A total of 7% of the country's population are senior citizens (45). Most of these senior citizens and many mid-aged people in the country have non-communicable disorders, including chronic obstructive pulmonary disease (11.9%), cardiac disorders (4.5%), diabetes (9.7%), and asthma (5.2%), and they are especially vulnerable to COVID-19 (46–49). Besides, there are around 1.3 to 1.5 million cancer patients in the country (50). Moreover, the prevalence of smoking is highest in Bangladesh among the South Asian countries (49). Studies have reported that people who smoke and have cancer have a higher risk of developing serious complications. Although there is still a dearth of understanding of the association between COVID-19 severity and cancer and smoking, these could likely be correlated (51, 52). In the case of an upsurge of people who belong to the vulnerable groups contracting COVID-19, they may require hospitalization and intensive care. Hence, ventilation supports in every hospital, clinic, and medical center is a must. The country has so far arranged only 112 beds across the country in intensive care units for patients with COVID-19 (53). The tech start-up and innovation companies emerging in the country should take it as a challenge to design a cheap but rapidly deployable mechanical ventilator device. All non-essential surgeries and hospital admissions should be canceled immediately to make sure the hospitals are not unnecessarily occupied. Hospitals can become a source of COVID-19 transmission, and it is advisable to decentralize healthcare services and, whenever possible, to provide care at home. Government rest houses and private hotels can be turned into emergency response healthcare facilities. Moreover, as a riverine country, Bangladesh has a huge water transport system. Large water vehicles, including steamers and launches, can be used as mobile healthcare facilities for the people who live in remote areas.

## Coping With Mental Stress Due to COVID-19

Fear and anxiety about the pandemic are causing overwhelming stress for everyone (54, 55). While receiving mixed messages piles up the stress, sharing the real facts and understanding the actual risk reduces the stress. Moreover, this helps the authorities to organize better and manage the crisis. Social activists, television and print media, social workers, and religious and political leaders should come forward to help in the dissemination of scientifically factual information on nCoV-2 and COVID-19 among the mass population of Bangladesh. For instance, the Imams (a Muslim leadership position) of each mosque could play a vital role in fighting this extraordinary crisis in Bangladesh (56). Together, the media personalities and political and religious leaders could help spread basic knowledge on COVID-19-related issues to the mass populace, especially the marginalized communities. Given the high level of illiteracy among the slum and village population, the dissemination of COVID-19-related basic knowledge would be the key to controlling the spread of the virus (57).

## Need for a Considerable Amount of Funds

Above all, Bangladesh must source a decent emergency support fund to help its workers, employers, parents, marginal people, and hosted refugees. It has already received fast-track support of USD 100 million from the World Bank; however, this is far from the actual amount needed for this country of 180 million people (58, 59). Additionally, the country has recently unveiled an economic stimulus package of ~8 billion USD to counter the adverse effects of the pandemic (34). The country may temporally postpone all non-essential developmental works and gather a modest amount of money to support its people in fighting this crisis. Also, top business organizations and international funders should come forward to help Bangladesh fight the COVID-19 challenge. Only a supportive and empathic collaborative effort can help the world, especially the low and lower-middle-income countries like Bangladesh, overcome this crisis.

## Conclusions

Preparedness is the key to addressing any health crisis, and so far, Bangladesh, as a lower-middle-income country, has numerous limitations in restricting the spread of the virus. While continuing the lockdown at any cost with more strict maintenance, the country has to expand its testing and healthcare facilities. It has to ensure a constant supply of PPE for healthcare workers. Above all, improvised and timely measures taken with proper coordination may help the country to fight the lethal virus. The Government will not be able to mitigate the situation alone (60); individual efforts from the citizens, direct involvement of the nation's public health experts, and international help are urgently needed. As the situation intensifies, the world is closely watching how Bangladesh will navigate this crisis.

## Author Contributions

MH conceived the study. SA wrote the first draft. MN commented on the draft and contributed to the writing of the manuscript. All authors approved the final version of the manuscript.

## Conflict of Interest

The authors declare that the research was conducted in the absence of any commercial or financial relationships that could be construed as a potential conflict of interest.
